# Dietary Soy Protein Isolate Attenuates Intestinal Immunoglobulin and Mucin Expression in Young Mice Compared with Casein

**DOI:** 10.3390/nu12092739

**Published:** 2020-09-08

**Authors:** Bin Zeng, Dongyang Wang, Hailong Wang, Ting Chen, Junyi Luo, Qianyun Xi, Jiajie Sun, Yongliang Zhang

**Affiliations:** Guangdong Provincial Key Laboratory of Animal Nutrition Control, National Engineering Research Center for Breeding Swine Industry, Guangdong Laboratory for Lingnan Modern Agriculture, College of Animal Science, South China Agricultural University, Guangzhou 510642, China; zengbin@stu.scau.edu.cn (B.Z.); wangdy@stu.scau.edu.cn (D.W.); wanghailong03@stu.scau.edu.cn (H.W.); allinchen@scau.edu.cn (T.C.); luojunyi@scau.edu.cn (J.L.); xqy0228@scau.edu.cn (Q.X.); jiajiesun@scau.edu.cn (J.S.)

**Keywords:** Soy protein isolate, casein, intestine, SIgA, mucin

## Abstract

Dietary protein sources have profound effects on children and young animals, and are important for the gut barrier function and immune resilience. Milk and soy are the main sources of protein for children and young animals after weaning. The objective of this study was to compare the effects of dairy and soy proteins on the intestinal barrier in early development. Weanling C57BL/6 mice were fed AIN-93G diets prepared with casein or soy protein isolate (SPI) for 21 days. Compared with those fed with the casein diet, mice fed with the SPI diet did not change their body weight and organ coefficients, but increased their feed intake and ratio of feed to gain. SPI lowered the level of luminal secretory immunoglobulin A (SIgA) and downregulated the levels of IL-4, IL-13, polymeric immunoglobulin receptor (*Pigr*), Janus kinase 1 (*Jak1*), signal transducer and activator of transcription 6 (*Stat6*), and transforming growth factor-β (*Tgfb*) in the mouse ileum. Western blotting of ileal proteins confirmed that SPI suppressed the activation of the JAK1/STAT6 signaling pathway. Furthermore, SPI attenuated intestinal mucin production, as demonstrated by the decreased numbers of intestinal goblet cells and the reduced relative expression levels of mucin 1 (*Muc1*), mucin 2 (*Muc2*), trefoil factor 3 (*Tff3*), glucose-regulated protein 94 (*Grp94*), and anterior gradient homolog 2 (*Agr2*). The results indicated that the SPI diet could attenuate mouse intestinal immunity, as demonstrated by decreased SIgA and mucin production in the intestine. Therefore, we suggest that our findings should be of consideration when SPI or casein are used as dietary protein sources.

## 1. Introduction

The diets consumed after weaning can have long-term effects on the development and health of infants. Infant formulas (IFs) can be defined as substitutes for breast milk after weaning. Bovine milk protein (with casein as the main ingredient) is the source of protein in most IFs. According to the applicable European regulation (EU, 2016), soy protein isolate (SPI) is allowed as a source of protein in IFs [1]. Although the use of soy protein-based formula is recommended only for infants who cannot receive milk-based products because of cow milk allergy, lactose intolerance or cultural/religious reasons [2], soy protein-based formulas in the United States account for nearly 25% of the formula market [3]. The main reasons for this are likely to be the rising global incidence of obesity and metabolic syndrome in both adults and children [4], for which soy foods are posited as irresistible health diets, and are described as being high in protein, but low in saturated fat and cholesterol [5]. Additional health benefits have been suggested to include anti-diabetic effects and protection against coronary heart disease, based on reductions in plasma cholesterol and triglycerides [6]. However, the potential health effects of soy foods in children and young animals remain highly controversial [7,8].

The gut is the core organ that comes into contact with food. Besides its central function in the digestion and absorption of nutrients, the intestine provides a robust mucosal barrier that exerts a protective function in the body [9]. The intestinal structure and function of children or young animals is incomplete. Importantly, during weaning, the sudden transition from breast milk to solid feed causes weaning stress that damages the intestinal mucosal barrier and results in an intestinal imbalance [10]. Intestinal diseases in childhood can lead to anemia, malnutrition, and impaired mental skills, which are hazardous to long-term life quality [11,12]. In livestock production, intestinal damage in young animals results in decreased growth rates and increased morbidity and mortality [13].

The gut is a multilayer system mainly consisting of a mucus layer produced by the goblet cells, followed by a monolayer of epithelial cells forming the epithelial tight junction [14]. The secretion of highly glycosylated mucins into the intestinal lumen by goblet cells creates the first line of defense against microbial encroachment. The most abundant one of these mucins, mucin 2 (MUC2), plays an essential part in the organization of intestinal mucous layers at the epithelial surface [15]. Mucosal-acquired immunity is an indispensable line of defense against the gut bacteria of the mucosa and is an essential component of the gut barrier function. Secretory immunoglobulin A (SIgA) is a primary protective factor for gut-acquired immunity and is transported from the basolateral surface to the mucosal surface by binding to the polymeric immunoglobulin receptor (pIgR) [16]. Dietary protein provides nutritional and functional components to the body, which may possess diverse properties and result in health effects in the intestinal tract. It has been reported that different dietary protein sources have different effects on regional gut growth and luminal growth factor bioactivity [17]. Rats fed with different protein diets showed a rapidly altered intestinal microbial composition [18]. The long-term supply of a particular diet of proteins leads to specific metabolic activities of the intestinal microbiota [19]. However, there is still a lack of sufficient and rigorous animal experiments to compare the effects of dairy and soy proteins on the intestinal barrier from weaning to puberty.

Based on current knowledge, we hypothesized that dietary supplementation with milk or soy source preparations have different effects on the host intestinal mucosal barrier during early development. Therefore, a casein-based diet and a soy protein isolate (SPI)-based diet were supplied as the sole dietary protein source for weanling C57BL/6 mice. The host growth, feed intake, and the intestinal mucosal barrier function were then examined. The results provided evidence that milk and soy proteins have different effects on intestinal health, and also provided important insights for the applications of milk and soy protein sources in children or young animal diets.

## 2. Materials and Method

### 2.1. Diets

The diets were custom-made by Guangdong Medical Laboratory Animal Center (Guangdong, China). The casein diet was formulated according to the AIN-93G formulation [20]. The SPI diet was made by replacement of casein with SPI (food grade, YUWANG GROUP products, Shandong, China, execution standard: GB/T20371-2016), and essential amino acids (Tagn Rusii products, US) were added to match the casein diet. The contents of crude protein and dry matter of SPI were adjusted to be consistent with those of the casein diet. Table 1 shows the measured amino acid content of casein and SPI. Table 2 shows the overall composition of the diets.

### 2.2. Animal Experiments

Sixteen male C57BL/6 weanling mice at 3 weeks of age were obtained from the Animal Experiment Center of Guangdong Province (permission number: SYXK [Yue] 2016-0136). The animals were randomly divided into two groups of eight mice each. Mice in the casein group and SPI group were fed with the casein diet and SPI diet, respectively. Mice were caged individually, housed in a room maintained under a photoperiod of 12/12 h (day/night), a temperature of 24 ± 2 °C, and a relative humidity of 60 ± 10% throughout the experimental period. In the following 21 days, all mice had free access to food and drinking water. During the experiment, the feed intake was measured every 3 days. Body weight was recorded each week. On day 22, the mice were sacrificed to obtain their organ weights and intestinal samples. The small intestine was excised and segmented. The luminal contents (mucosal scrapping) of the ileum were frozen in liquid nitrogen and stored at −80 °C for use in enzyme-linked immunosorbent assay (ELISA)–SIgA analyses. Mice ileum tissues (0.5 cm ×3) were frozen in liquid nitrogen and stored at −80 °C for use in ELISA-interleukin (IL)-4 and IL-13, quantitative real-time PCR (qRT-PCR), and Western blotting analyses. Another ileum tissue sample (1 cm) was fixed in 4% paraformaldehyde at room temperature for periodic acid–Schiff (PAS) staining and immunofluorescence analysis. The animal study was reviewed and approved by the Institutional Animal Care and Use Committee of South China Agricultural University, China. All animal experimentation complied with the laboratory animal management and welfare regulations approved by the Standing Committee of Guangdong People’s Congress (Guangzhou), China. Ethical code number: SCAU-AEC-2010-0416.

### 2.3. Enzyme-Linked Immunosorbent Assay

For SIgA quantification, the frozen ileal luminal contents were diluted with phosphate-buffered saline (PBS) containing phenylmethylsulfonyl fluoride (PMSF) (1 mM) at a ratio of 1:10 (*w*/*v*), homogenized, and centrifuged (12,000× *g*, 5 min, 4 °C). The resulting supernatant was collected to detect the levels of SIgA using an ELISA kit. For IL-4 and IL-13 quantification, intestinal proteins were lysed by grinding the ileal tissues in lysis buffer containing PMSF (1 mM), and centrifuged (12,000× *g*, 5 min, 4 °C). The resulting supernatant was collected to detect the protein concentration using bicinchoninic acid (BCA) protein assays (Thermo Scientific Technologies, Wilmington, DE, USA). The protein concentrations were adjusted to the same level and the samples were applied to analyze the intestinal IL-4 and IL-13 levels using the corresponding ELISA kits. All assays were performed according to the manufacturer’s protocols.

### 2.4. Gene Expression Analysis Using qRT-PCR

Total RNA was extracted from ileum tissue using the TRIzol reagent (Invitrogen, Carlsbad, CA, USA) according to the manufacturer’s instructions, and cDNA was synthesized using a reverse transcription kit (PrimeScript RT Reagent Kit with gDNA Eraser; Takara, Dalian, China) using 1 μg of total RNA, according to the manufacturer’s instructions. The cDNA was diluted with 5-fold nuclease-free H_2_O, and the qRT-PCR was performed using a CFX96 Touch™ Optics Module instrument (BIO-RAD, Hercules, CA, USA) in a 20 μL reaction containing 2 μL of cDNA, 5 mM of each primer, and 10 μL of 2× SYBR Green Real-time qPCR Master Mix reagents (Promega, Madison, WI, USA). The relative expression of mRNAs was normalized to *Actb* (β-actin) levels, using the 2^−ΔΔCt^ method. The primers used for qRT-PCR are shown in Table 3.

### 2.5. Western Blotting Analysis

Total protein was extracted from 100 mg of ileum tissue using 300 μL of radio immunoprecipitation assay (RIPA) lysis buffer that contained 1 mM PMSF and protein phosphatase inhibitor complex (Biosino Bio-Technology and Science Inc., Beijing, China). The sample was then centrifuged at 12,000× *g* at 4 °C for 5 min, and the supernatant was used for further analysis. The total protein concentration was determined using BCA protein assays (Thermo Scientific Technologies). Protein samples (25 μg) were separated by 10% SDS-PAGE, then transferred to 0.45 mm polyvinylidene fluoride (PVDF) membranes (Millipore, Bedford, MA, USA). After blocking with 5% skim milk for 2 h, the membranes were incubated overnight at 4 °C with specific antibodies. The following antibodies were used: anti-Janus kinase-1 (JAK1) (bs-1439R, Bioss, Beijing, China), anti-phospho (p)-JAK1 (Tyr1034 + Tyr1035, bs-3238R, Bioss, China), anti- signal transducer and activator of transcription 6 (STAT6) (380957, ZEN BIO, China), anti-p-STAT6 (Tyr641, ab263947, Abcam, Cambridge, MA, USA), anti-pIgR (AF2800-SP, R&D Systems, Minneapolis, MN, USA), anti-TGFβ1 (bs-4538R, Bioss, China), and anti-β-actin (AP0060, Bioworld Technology Inc., Bloomington, MN, USA). They were then incubated for 1 h at room temperature with horseradish peroxidase (HRP)-conjugated corresponding secondary antibodies. The immunoreactive protein bands were visualized using FluorChem M (ProteinSimple, San Jose, CA, USA). ImageJ software (NIH, Bethesda, MD, USA) was used for gray scan analysis.

### 2.6. Periodic Acid-Schiff Staining

Fixed ileum tissues were embedded in paraffin and 5-μm sections were prepared. Sections were stained with PAS/Hematoxylin blue. Images were acquired under a light microscope (Nikon, Tokyo, Japan) at a magnification of 40×. Positively stained cells were counted in all villi for each tissue section.

### 2.7. Immunofluorescence

Fixed ileum tissues were embedded in paraffin and 5-μm sections were prepared. The sections were incubated overnight in rabbit anti-MUC2 (NBP1-31231, Novus Biologicals, Littleton, CO, USA) at 4 °C. Then, the sections were washed with PBS three times and incubated in fluorescein isothiocyanate (FITC)-conjugated secondary antibodies (bs-0295G, Bioss) for 1 h at room temperature in the dark. For visualization of cell nuclei, 4′,6-diamidino-2-phenylindole (DAPI) (H-1200, Vector Laboratories) was added. Images were captured under a fluorescent microscope (Nikon) at a magnification of 200×. Positively stained cells were counted in all villi for each tissue section.

### 2.8. Statistical Analysis

All data are expressed as means ± the standard error of the mean (SEM). Data were analyzed using *t*-tests (SPSS 17.0, IMB Corp., Armonk, NY, USA). *p* < 0.05 was considered statistically significant.

## 3. Results

### 3.1. Body Weight and Feed Intake

**Figure 1:**
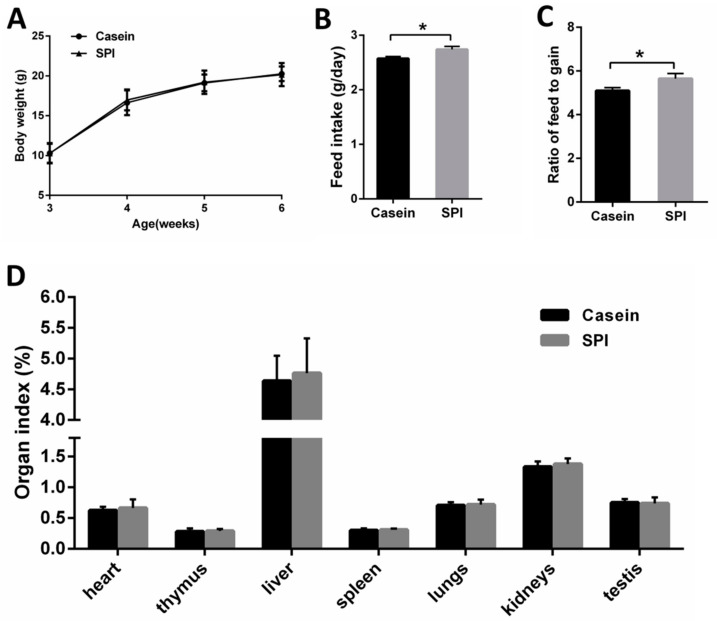
Growth performance of mice fed casein or soy protein isolate (SPI) diets. (**A**) Body weight. (**B**) Feed intake. (**C**) Ratio of feed to gain. (**D**) Organ index. All data are presented as means ± SEM, *n* = 8. * *p* < 0.05. Statistical significance was calculated using *t*-tests.

First, we investigated whether the two protein diets had different effects on the body weight and feed intake of the mice. The results showed that body weights of the mice were not different between the casein and SPI groups throughout the experiment (*p* > 0.05, Figure 1A). However, compared with the casein diet, feeding mice with the SPI diet increased the daily feed intake and the ratio of feed to gain (*p* < 0.05, Figure 1B,C). We also measured the organ weights of all mice at the end of the feeding study, and the results showed that there was no significant difference in the organ coefficients between the casein and SPI groups (*p* > 0.05, Figure 1D).

### 3.2. SPI Reduces Intestinal Secretory IgA (SIgA) and Th2 Cytokine Levels

**Figure 2:**
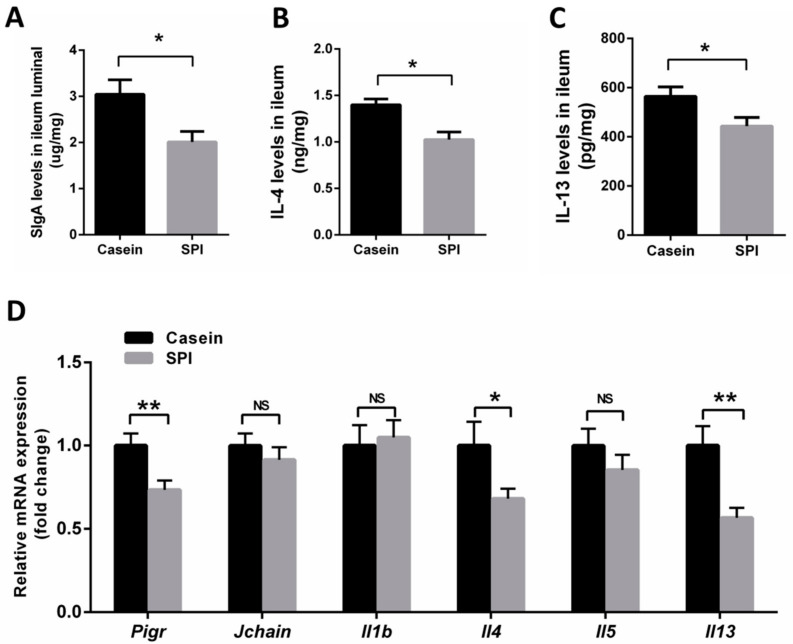
Effect of SPI on SIgA, IL-4, IL-13 and SIgA-associated mRNA levels in mouse ileum tissue. (**A**–**C**) ELISA analysis of SIgA, IL-4, and IL-13 levels in mouse ileum tissue, respectively (*n* = 7). (**D**) qRT-PCR analysis of *Pigr, Jchain, Il1β, Il4, Il5* and *Il13* mRNA expression in mouse ileum tissue (normalized against *Actb* (β-actin); *n* = 8). All data are presented as means ± SEM. NS, not significant (*p* > 0.05); * *p* < 0.05; ** *p* < 0.01. Statistical significance was calculated using *t*-tests.

As mentioned above, little is known about the effects of the two protein diets on intestinal barrier function in mice. SIgA plays an important role in the homeostatic regulation of intestinal mucosal immunity that protects the internal body from external enteric toxins and pathogenic microorganisms. Here, we examined SIgA levels in the ileum via ELISA. We observed significantly decreased SIgA levels in mice receiving the SPI diet compared with those in mice receiving the casein diet (*p* < 0.05, Figure 2A). Previous studies reported that Th2 cytokines are inducers of intestinal SIgA production [21]. Hence, we quantified the abundance of IL-4 and IL-13 proteins in the ileum using ELISA. The results showed that mice fed with SPI diets had lower IL-4 and IL-13 proteins levels than the casein-fed mice (*p* < 0.05, Figure 2B,C). We also investigated the expression levels of the mRNAs encoding J-chain (*Jchain*), polymeric immunoglobulin receptor (*Pigr*, the core components of SIgA), and related cytokines using qRT-PCR. The mRNA expression levels of *Jchain*, *Il1b* and *Il5* in the ileum were not significantly different between the casein and SPI groups (*p* > 0.05, Figure 2D). Interestingly, the mRNA expression levels of *Pigr*, *Il4* and *Il13* in the ileum were downregulated in the SPI group (*p* < 0.05, Figure 2D). These data suggested that compared with casein, SPI reduces SIgA production in the mouse ileum, which is associated with decreased levels of pIgR, IL-4, and IL-13.

### 3.3. SPI Suppresses the Intestinal JAK1/STAT6 Signaling Pathway

**Figure 3:**
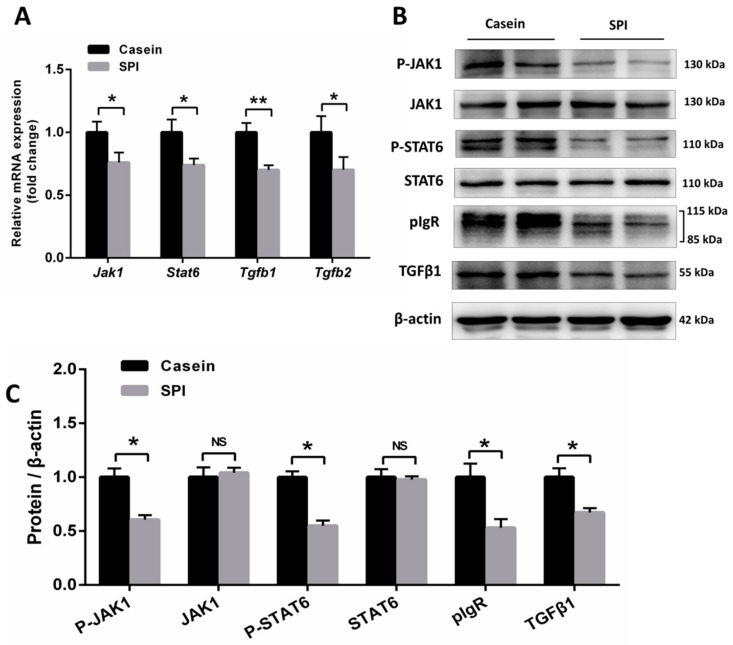
Effect of SPI on JAK1-STAT6 signaling and TGFβ levels in mouse ileum tissue. (**A**) qRT-PCR analysis of *Jak1*, *Stat6*, *Tgfb1*, and *Tgfb2* mRNA expression levels in mouse ileum tissue (normalized against *Actb* (β-actin); *n* = 8). (**B**) Western blotting analysis of JAK1, *p*-JAK1, STAT6, *p*-STAT6, pIgR, and TGFβ1 protein levels in mouse ileum tissue (*n* = 6). (**C**) The statistical analyses of the Western blotting results. Results are normalized to β-actin (*n* = 6). All data are presented as means ± SEM. NS, not significant (*p* > 0.05); * *p* < 0.05; ** *p* < 0.01. Statistical significance was calculated using *t*-tests.

Previous work has demonstrated that pIgR is regulated by the JAK-STAT pathway, and cytokines IL-4 and IL-13 are associated with increased JAK1 and STAT6 phosphorylation [22]. Thus, we analyzed the expression levels of *Jak1* and *Stat6* in the ileum. The results showed that the mRNA expression levels of *Jak1* and *Stat6* were decreased in the SPI group mice compared with those in the casein group (*p* < 0.05, Figure 3A). Western blotting analysis revealed that the levels of pIgR, *p*-JAK1, and *p*-STAT6 proteins in the ileal tissues of mice from the SPI group were significantly lower than those in the casein group (*p* < 0.05, Figure 3B,C). Moreover, *Tgfb* mRNA levels were reduced in the SPI group mice relative to those in the casein group (*p* < 0.05, Figure 3A), which was further confirmed using Western blotting (*p* < 0.05, Figure 3B,C). These results indicated that, compared with the casein diet, the SPI diet reduces SIgA levels in the mouse ileum by suppressing the JAK1/STAT6-pIgR signaling pathway.

### 3.4. SPI Reduces Intestinal Mucin Production

**Figure 4:**
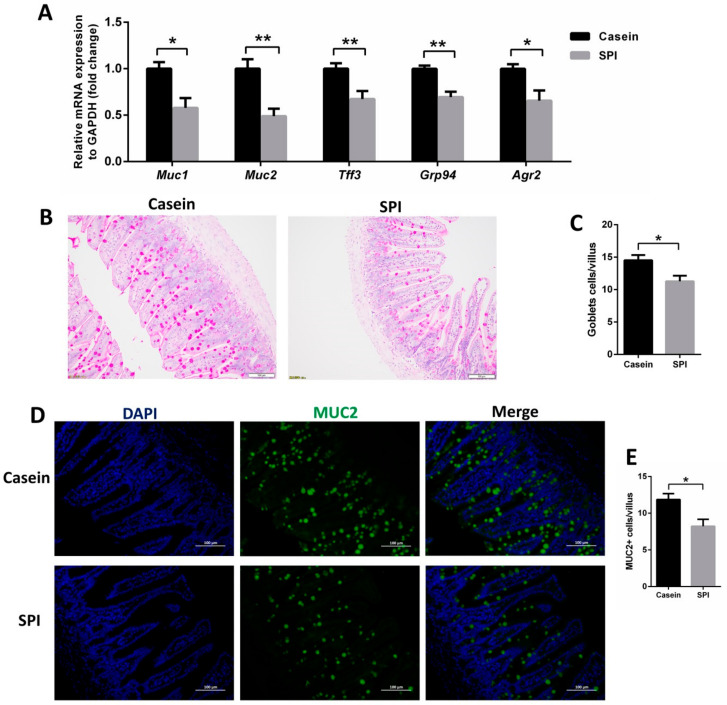
Effect of SPI on mucin production in mouse ileum tissue. (**A**) qRT-PCR analysis of *Muc1*, *Muc2*, *Tff3*, *Grp94*, and *Agr2* mRNA expression levels in mouse ileum tissue (normalized against *Actb* (β-actin); *n* = 8). (**B**,**C**) Representative PAS-stained histomicrographs and corresponding quantification of goblets cell per villus in the ileum tissue (*n* = 6). (**D,E**) Representative micrographs for MUC2 staining and corresponding quantification of MUC2^+^ goblet cells per villus in the ileum tissue (*n* = 6). All data are presented as means ± SEM. * *p* < 0.05; ** *p* < 0.01. Statistical significance was calculated using *t*-tests.

We also compared the effect of casein and SPI on intestinal mucin production. The ileal mRNA levels of mucin 1 (*Muc1*), mucin 2 (*Muc2*), trefoil factor 3 (*Tff3*), glucose-regulated protein 94 (*Grp94*), and anterior gradient homolog 2 (*Agr2*), as measured using qRT-PCR, were significantly decreased in SPI diet-fed mice compared with those in the casein diet-fed mice (*p* < 0.05, Figure 4A). PAS staining analysis revealed that the number of goblet cells per villus of the ileum was significantly reduced in the SPI diet group compared with that in the casein diet group (*p* < 0.05, Figure 4B,C). Similarly, immunofluorescence analysis revealed that the number of MUC2-positive cells per villus was decreased in the SPI group relative to that in the casein diet group (*p* < 0.05, Figure 4D,E). These data suggested that the SPI diet also reduces intestinal mucin production compared with that of the casein diet.

## 4. Discussion

Dairy and soy products are the main sources of protein for children and young animals after weaning. In this study, we compared the differences in intestinal immunoglobulin and mucin between mice fed casein and SPI diets. From a nutritional perspective, casein is a high-quality protein, and it is therefore used as a protein source in the well-balanced semisynthetic AIN-93G diet [20]. In addition, the original source of casein is cow’s milk, which is considered to be mostly consistent with infant formula. We used weaned C57BL/6 mice as an animal model, and the casein-based semisynthetic diet (AIN-93G) was used as the reference diet. The AIN-93 diet is the global standard for a purified rodent diet proposed by the American Institute of Nutrition (AIN) and is considered as the “gold standard” in nutrition research [23]. Moreover, to remove potential confounding factors, careful measures were taken to ensure the same protein intake in the two groups. The concentrations of dry matter, crude protein, and essential amino acids in two protein sources were adjusted to be consistent in the present study. Infant formula (soy protein-based or milk protein-based) is usually used as a sole-source food for infants and is consumed by infants for long periods of time [24]. The content of essential amino acids in soy protein-based infant formula is usually consistent with that in milk protein-based infant formula and meets the standard of infant formula [25]. Therefore, we believe the findings in our study will also be relevant to human nutrition research.

Our results showed that compared with feeding casein protein, feeding mice with the SPI protein diet significantly increased the feed intake and ratio of feed to gain of the mice, but did not change their body weight and organ coefficients. This indicated that SPI inhibited the feed conversion rate. Similar results were observed in previous studies [23,26], in which growing rats were fed casein or SPI AIN-93G diets. In those studies, however, the decline in feed conversion efficiency in the SPI rats was not caused by the increased feed intake, but by the decreased weight gain. They believed that the weight gain inhibition effects of dietary SPI on the young rats were attributed to essential amino acid limitation (methionine) in the SPI source, a fact that we have also found in the methionine concentration in SPI (0.73%), which is much lower than that in casein (2.29%), as shown in Table 1. Their results also showed that the total plasma amino acid concentrations in the young rats fed the SPI diet were significantly reduced (by 28.3%), among which the essential amino acid concentrations were especially reduced (by 37.7%) [23]. In the present study, we did not observe a decrease in body weight in the SPI diet-fed mice, which might have resulted from the concentrations of essential amino acids in two protein sources having been adjusted to be consistent. Wróblewska et al. [27] and Song et al. [23] reported that SPI decreases the protein efficiency ratio and increases the concentration of N in urine and plasma. Moreover, soy proteins led to a lower level of gastrointestinal digestion than dairy proteins during in vitro infant digestion [28], which may trigger a reduction in the feed conversion rate.

In this work, we focused on the effects of casein and soy protein isolate on the intestinal barrier in mice. To the best of our knowledge, this is the first report to demonstrate that feeding SPI results in the suppression of SIgA and mucin in mouse intestines compared with feeding casein. We observed that the SPI diet decreased SIgA levels in the mouse ileum. SIgA has the capacity to directly quench bacterial virulence factors and to promote the clearance of ls, which would provide opportunities for pathogens to access epithelial receptors and pass through antigens and pathogenic microorganisms from the intestinal lumen [29]. Reduced intestinal SIgA levels in the intestinal epithelia lead to intestinal and other diseases. The production and secretion of intestinal IgA are complex and regulated by many factors [21]. Mice fed with the SPI diet had lower IL-4, IL-13, p-JAK1, p-STAT6, and pIgR levels than the casein-fed mice. Cytokines IL-4 and IL-13 are stimulators of intestinal SIgA production [21,30]. A previous study demonstrated that IL-4 and IL-13 induce tyrosine phosphorylation and activation of STAT-6 [31]. The secretion of SIgA across the intestinal epithelia via pIgR is regulated by the JAK1-STAT6 pathway [22]. We found that SPI significantly reduced the levels of the Th2-associated cytokines, IL-4 and IL-13, in intestinal tissues, further resulting in reduced levels of phosphorylated JAK1 and STAT6, which ultimately inhibited the expression of the IgA transport protein pIgR and lowered the luminal SIgA level. The TGFβ level in the mouse ileum decreased in response to the SPI diet, which also contributed to suppressing intestinal SIgA secretion. TGFβ is arguably the most important factor in directing B cells toward IgA differentiation [32]. TGFβ can be produced by various cell types, including epithelial cells, dendritic cells, and T cells [21]. IgA production levels decreased remarkably in TGFβ1 knockout mice [33]. Compared with the mice fed the casein diet, the lack of corresponding cytokines in the SPI-fed mice might be responsible for this effect [34]. Our study also found that, compared with casein, SPI significantly reduced the numbers of goblet cells and MUC2-positive cells, and the expression levels of *Muc2*, *Tff3*, *Grp94*, and *Agr2* in the ileum. These results were consistent with a previous report [35], in which a soy protein diet decreased Mucin and TFF3 levels in the colon of dextran sodium sulfate-treated C57BL/6 mice. Goblet cells produce mucins, which constitute the mucus layer overlying the gut surface epithelium, and play an essential role in the protection of the intestinal tract from injury [36]. MUC2 and TFF3 are the principal components in the small intestine responsible for construction of the mucus barrier [37]. GRP94 is the most abundant protein within the endoplasmic reticulum lumen, and has a critical link to goblet cell and gut barrier function [38]. AGR2 is present within the endoplasmic reticulum of intestinal secretory epithelial cells and is essential for in vivo production of intestinal MUC2 [39]. This series of reduction altogether could lead to damage in the intestinal barrier.

SPI is a food stuff comprising a complex mixture, i.e., proteins, peptides, and over 100 phytochemicals, all of which might have biological activities [40]. It remains unclear which component(s) of SPI are responsible for the observed effects on the intestinal barrier in weaned mice. Thus, further studies of the potential mechanisms and bioactive components are warranted. It has been reported that branched chain amino acids (BCAA) play an important role in the intestinal barrier and immune function in animals [41]. Soy protein contains less BCAA than dairy protein [42]. This may also be one of the reasons for the results in this study. Recent evidence suggests that microRNAs in food can be absorbed into the circulatory system and organs of the host, where they regulate gene expressions and biological processes [43,44]. Plant (maize) miRNA-156 inhibits intestine cell proliferation by targeting the Wnt/β-catenin pathway [45]. Soybean-derived miRNA-159a specifically inhibits proliferation and stimulates apoptosis of Caco-2 cells [46]. Interestingly, highly abundant miRNAs in bovine milk, including miR-148a and the let7 family [47], have been reported to play an important regulatory role in intestinal mucosal immunity [48]. Therefore, it is important to analyze the content and biological effects of microRNAs in SPI and casein on animal intestines in future research. Food-derived bioactive peptides play an important role in intestinal barrier function [49]. It would be meaningful to explore the differences in peptide composition in the intestines and even in the fecal samples. In addition, the composition and metabolic activity of the gut microbiota could be modulated by dietary proteins. These changes of microbiota could affect the gut barrier and the immune system by regulating the secretion of metabolites and gene expression in relevant signaling pathways [10]. Further studies to determine the importance of gut bacteria in these animals could include explorations into intestinal microbial species and short chain fatty acids (SCFAs), or the use of germ-free animals. There were still some limitations in this study. The sex of mice could affect some parts of the responses to dietary proteins. Since we did not include female mice in this study, it is difficult to tell the effects of these proteins on the female mice. As we examined ileum tissues, we cannot determine the changes occurring in the whole gut tract. Therefore, more studies are needed to comprehensively understand the changes in the intestinal barrier caused by milk and soy protein.

## 5. Conclusions

In conclusion, this study has demonstrated that, compared with casein, SPI reduces intestinal SIgA and mucin production, and induces negative effects on the feed conversion rate in young mice. Our findings provide new data that should be taken into account when soy protein is considered to replace dairy protein in the diets of children or young animals.

## Figures and Tables

**Table 1:** Amino acid composition of casein and soy protein isolate (SPI), on a dry matter basis.

Item (%)	Casein	SPI
Asp	6.71	10.30
Thr	3.76	3.37
Ser	4.38	4.19
Glu	18.78	16.60
Gly	1.74	3.73
Ala	2.90	3.88
Val	5.65	4.31
Met	2.29	0.73
Ile	4.53	4.14
Leu	8.50	7.02
Tyr	4.74	3.26
Phe	4.59	4.64
Lys	6.77	5.66
His	2.37	2.29
Arg	3.01	6.68
Pro	8.90	4.57
Trp	1.10	1.12

**Table 2:** Composition of AIN-93G-based casein and soy protein isolate (SPI) diets.

Ingredient (g/kg)	Casein Diet	SPI Diet
Cornstarch	397.468	397.468
Casein (≥85% protein)	200.000	0
Soy protein isolate *	0	200.000
Dextrinized cornstarch	132.000	132.000
Sucrose	100.000	100.000
Soybean oil (no additives)	70.000	70.000
Fiber	50.000	50.000
Mineral mix (AIN-93G-MX)	35.000	35.000
Vitamin mix (AIN-93-VX)	10.000	10.000
L-Cystine	3.000	3.000
Choline bitartrate (41.1% choline)	2.500	2.500
Tert-butylhydroquinone	0.014	0.014

* The essential amino acids were added to match the casein levels.

**Table 3:** Primers for qRT-PCR.

Name	Forward (5′-3′)	Reverse (5′-3′)
β-actin	TGCTGTCCCTGTATGCCTCT	CTTTGATGTCACGCACGATTT
J-chain	GAACTTTGTATACCATTTGTCAGACG	CTGGGTGGCAGTAACAACCT
pIgR	AGTAACCGAGGCCTGTCCTT	GTCACTCGGCAACTCAGGA
TGFβ1	ATTCCTGGCGTTACCTTGG	AGCCCTGTATTCCGTCTCCT
TGFβ2	ACATCCACACGCACACTCAT	AAGGGACGAGACGAGAAGGT
JAK1	AGTGCAGTATCTCTCCTCTCTG	GATTCGGTTCGGAGCGTACC
STAT6	ATCTTCAACGACAACAGCCTCA	GGAGAAGGCTAGTGACATATTG
IL-1β	TTGAAGTTGACGGACCCCA	CCACAGCCACAATGAGTGATAC
IL-4	AACGAGGTCACAGGAGAAGG	TGGAAGCCCTACAGACAAGC
IL-5	CCCTCATCCTCTTCGTTGC	ATCCTCCTGCGTCCATCTG
IL-13	CTTGCTTGCCTTGGTGGTC	GGGAGTCTGGTCTTGTGTGA
MUC1	CCTTCAGTGCCAAGTCAATAC	TCCCCAGAAAATCTCCGTT
MUC2	ATGCCCACCTCCTCAAAGAC	GTAGTTTCCGTTGGAACAGTGAA
TFF3	GCTAATGCTGTTGGTGGTCC	GGTTGTTACACTGCTCCGATG
GRP94	GGTGTTGTGGATTCCGATG	GAAGTTTAGCAAGCCGTGTT
AGR2	CTGTTGCTTGTCTTGGATCTGT	GGAGCCAAAAAGGACCCAAAG

## Abbreviations

PAS	Periodic Acid–Schiff
SPI	Soy protein isolate
SIgA	Secretory immunoglobulin A
IL-1β	Interleukin-1β
IL-4	Interleukin-4
IL-5	Interleukin-5
IL-13	Interleukin-13
pIgR	Polymeric immunoglobulin receptor
Jak1	Janus kinase 1
Stat6	Signal transducer and activator of transcription 6
TGFβ	Transforming growth factor-β
Muc1	Mucin 1
Muc2	Mucin 2
Tff3	Trefoil factor 3
Grp94	Glucose-regulated protein 94
Agr2	Anterior gradient homolog 2

## References

[1] Roux L.L., Mejean S., Chacon R., Lopez C., Dupont D., Deglaire A., Nau F., Jeantet R. (2020). Plant proteins partially replacing dairy proteins greatly influence infant formula functionalities. LWT Food Sci. Technol..

[2] Li H., Zhu K., Zhou H., Peng W., Guo X. (2013). Comparative Study about Some Physical Properties, In vitro Digestibility and Immunoreactivity of Soybean Protein Isolate for Infant Formula. Plant Foods Hum. Nutr..

[3] Bhatia J., Greer F.R. (2008). Use of Soy Protein-Based Formulas in Infant Feeding. Pediatrics.

[4] Flynn M.A.T., Mcneil D.A., Maloff B., Mutasingwa D., Wu M., Ford C., Tough S. (2006). Reducing obesity and related chronic disease risk in children and youth: A synthesis of evidence with ‘best practice’ recommendations. Obes. Rev..

[5] Uddin M.N., KanikaMitra D., Rahman M.M., Abdullah A., Haque D.M.Z. (2016). Evaluation of proximate, determination of minerals and chromatographic quantification of water soluble vitamin in newly developed soy protein isolate. J. Biosci..

[6] Ronis M.J., Chen Y., Badeaux J., Badger T.M. (2009). Dietary soy protein isolate attenuates metabolic syndrome in rats via effects on PPAR, LXR, and SREBP signaling. J. Nutr..

[7] Clarkson T.B. (2002). Soy, soy phytoestrogens and cardiovascular disease. J. Nutr..

[8] Badger T.M., Ronis M.J.J., Hakkak R., Rowlands J.C., Korourian S. (2002). The Health Consequences of Early Soy Consumption. J. Nutr..

[9] Damiano S., Sasso A., De Felice B., Di Gregorio I., La Rosa G., Lupoli G.A., Belfiore A., Mondola P., Santillo M. (2018). Quercetin increases MUC2 and MUC5AC gene expression and secretion in intestinal goblet cell-like LS174T via PLC/PKCα/ERK1-2 pathway. Front. Physiol..

[10] Ma N., Tian Y., Wu Y., Ma X. (2017). Contributions of the interaction between dietary protein and gut microbiota to intestinal health. Curr. Protein Pept. Sci..

[11] Hukkinen M., Merrassalmio L., Pakarinen M.P. (2018). Health-related quality of life and neurodevelopmental outcomes among children with intestinal failure. Semin. Pediatr. Surg..

[12] Elsayed N.M., Ramadan M.E. (2017). The Impact of Intestinal Parasitic Infections on the Health Status of Children: An Overview. J. Pediatr. Infect. Dis..

[13] Ji F.J., Wang L.X., Yang H., Hu A., Yin Y. (2019). Review: The roles and functions of glutamine on intestinal health and performance of weaning pigs. Animal.

[14] Turner J.R. (2009). Intestinal mucosal barrier function in health and disease. Nat. Rev. Immunol..

[15] Peterson L.W., Artis D. (2014). Intestinal epithelial cells: Regulators of barrier function and immune homeostasis. Nat. Rev. Immunol..

[16] Sun H., Bi J., Lei Q., Wan X., Jiang T., Wu C., Wang X. (2018). Partial enteral nutrition increases intestinal sIgA levels in mice undergoing parenteral nutrition in a dose-dependent manner. Int. J. Surg..

[17] Marchbank T., Mandir N., Calnan D., Goodlad R.A., Podas T., Playford R.J. (2018). Specific protein supplementation using soya, casein or whey differentially affects regional gut growth and luminal growth factor bioactivity in rats; implications for the treatment of gut injury and stimulating repair. Food Funct..

[18] Zhao F., Huang Z., Zhou G., Li H., Xu X., Li C. (2017). Dietary proteins rapidly altered the microbial composition in rat caecum. Curr. Microbiol..

[19] Graf D., Di Cagno R., Fåk F., Flint H.J., Nyman M., Saarela M., Watzl B. (2015). Contribution of diet to the composition of the human gut microbiota. Microb. Ecol. Health Dis..

[20] Reeves P.G., Nielsen F.H., Fahey G.C. (1993). AIN-93 Purified Diets for Laboratory Rodents: Final Report of the American Institute of Nutrition ad Hoc Writing Committee on the Reformulation of the AIN-76A Rodent Diet. J. Nutr..

[21] Pabst O. (2012). New concepts in the generation and functions of IgA. Nat. Reviews Immunol..

[22] Heneghan A.F., Pierre J.F., Kudsk K.A. (2013). JAK-STAT and intestinal mucosal immunology. Jak Stat.

[23] Song S., Hua C., Zhao F., Li M., Fu Q., Hooiveld G.J., Muller M., Li C., Zhou G. (2018). Purified dietary red and white meat proteins show beneficial effects on growth and metabolism of young rats compared to casein and soy protein. J. Agric. Food Chem..

[24] FAQ Expert Consultation (2013). Dietary protein quality evaluation in human nutrition. FAO Food Nutr. Pap..

[25] Agostoni C., Agostoni C., Axelsson I., Goulet O., Koletzko B., Michaelsen K.F., Puntis J., Rieu D., Rigo J., Shamir R. (2006). Soy protein infant formulae and follow-on formulae: A commentary by the ESPGHAN Committee on Nutrition. J. Pediatr. Gastroenterol. Nutr..

[26] Song S., Hooiveld G.J., Li M., Zhao F., Zhang W., Xu X., Muller M., Li C., Zhou G. (2016). Dietary soy and meat proteins induce distinct physiological and gene expression changes in rats. Sci. Rep..

[27] Wróblewska B., Juśkiewicz J., Kroplewski B., Jurgoński A., Wasilewska E., Złotkowska D., Markiewicz L. (2018). The effects of whey and soy proteins on growth performance, gastrointestinal digestion, and selected physiological responses in rats. Food Funct..

[28] Nguyen T.T.P., Bhandari B., Cichero J., Prakash S. (2015). Gastrointestinal digestion of dairy and soy proteins in infant formulas: An in vitro study. Food Res. Int..

[29] Mantis N.J., Rol N., Corthésy B. (2011). Secretory IgA’s complex roles in immunity and mucosal homeostasis in the gut. Mucosal Immunol..

[30] Wu M., Xiao H., Liu G., Chen S., Tan B., Ren W., Bazer F.W., Wu G., Yin Y. (2016). Glutamine promotes intestinal SIgA secretion through intestinal microbiota and IL-13. Mol. Nutr. Food Res..

[31] Heneghan A.F., Pierre J.F., Kudsk K.A. (2013). IL-25 improves IgA levels during parenteral nutrition through the JAK-STAT pathway. Ann. Surg..

[32] Stavnezer J., Kang J. (2009). The surprising discovery that TGFβ specifically induces the IgA class switch. J. Immunol..

[33] Van Ginkel F.W., Wahl S.M., Kearney J.F., Kweon M.N., Fujihashi K., Burrows P.D., Kiyono H., Mcghee J.R. (1999). Partial IgA-deficiency with increased Th2-type cytokines in TGF-beta 1 knockout mice. J. Immunol..

[34] Konstantinou G.N., Bencharitiwong R., Grishin A., Caubet J.C., Bardina L., Sicherer S.H., Sampson H.A., Nowak-Węgrzyn A. (2014). The role of casein-specific IgA and TGF-β in children with food protein-induced enterocolitis syndrome to milk. Pediatr. Allergy Immunol..

[35] Jiang H., Przybyszewski J., Mitra D., Becker C., Brehm-Stecher B., Tentinger A., MacDonald R.S. (2011). Soy protein diet, but not Lactobacillus rhamnosus GG, decreases mucin-1, trefoil factor-3, and tumor necrosis factor-α in colon of dextran sodium sulfate-treated C57BL/6 mice. J. Nutr..

[36] Johansson M.E., Sjövall H., Hansson G.C. (2013). The gastrointestinal mucus system in health and disease. Nat. Rev. Gastroenterol. Hepatol..

[37] Matsuo K., Ota H., Akamatsu T., Sugiyama A., Katsuyama T. (1997). Histochemistry of the surface mucous gel layer of the human colon. Gut.

[38] Liu B., Staron M., Hong F., Wu B.X., Sun S., Morales C., Crosson C.E., Tomlinson S., Kim I., Wu D. (2013). Essential roles of grp94 in gut homeostasis via chaperoning canonical Wnt pathway. Proc. Natl. Acad. Sci. USA.

[39] Park S.-W., Zhen G., Verhaeghe C., Nakagami Y., Nguyenvu L.T., Barczak A.J., Killeen N., Erle D.J. (2009). The protein disulfide isomerase AGR2 is essential for production of intestinal mucus. Proc. Natl. Acad. Sci. USA.

[40] Ronis M.J., Gomez-Acevedo H., Shankar K., Sharma N., Blackburn M., Singhal R., Mercer K.E., Badger T.M. (2018). Soy protein isolate feeding does not result in reproductive toxicity in the pre-pubertal rat testis. Exp. Biol. Med..

[41] Zhang S., Zeng X., Ren M., Mao X., Qiao S. (2017). Novel metabolic and physiological functions of branched chain amino acids: A review. J. Anim. Sci. Biotechnol..

[42] Kalman D.S. (2014). Amino Acid Composition of an Organic Brown Rice Protein Concentrate and Isolate Compared to Soy and Whey Concentrates and Isolates. Foods.

[43] Zhang L., Chen T., Yin Y., Zhang C.-Y., Zhang Y.-L. (2019). Dietary microRNA-A Novel Functional Component of Food. Adv. Nutr..

[44] Li J., Lei L., Ye F., Zhou Y., Chang H., Zhao G. (2019). Nutritive implications of dietary microRNAs: Facts, controversies, and perspectives. Food Funct..

[45] Li M., Chen T., Wang R., Luo J.-Y., He J.-J., Ye R.-S., Xie M.-Y., Xi Q.-Y., Jiang Q.-Y., Sun J.-J. (2019). Plant MIR156 regulates intestinal growth in mammals by targeting the Wnt/beta-catenin pathway. Am. J. Physiol. Cell Physiol..

[46] Liu J., Wang F., Weng Z., Sui X., Fang Y., Tang X., Shen X. (2020). Soybean-derived miRNAs specifically inhibit proliferation and stimulate apoptosis of human colonic Caco-2 cancer cells but not normal mucosal cells in culture. Genomics.

[47] Izumi H., Kosaka N., Shimizu T., Sekine K., Ochiya T., Takase M. (2012). Bovine milk contains microRNA and messenger RNA that are stable under degradative conditions. J. Dairy Sci..

[48] Park E.J., Shimaoka M., Kiyono H. (2017). MicroRNA-mediated dynamic control of mucosal immunity. Int. Immunol..

[49] Martinez-Augustin O., Rivero-Gutierrez B., Mascaraque C., Sanchez de Medina F. (2014). Food Derived Bioactive Peptides and Intestinal Barrier Function. Int. J. Mol. Sci..

